# An Engineering Approach to Extending Lifespan in *C. elegans*

**DOI:** 10.1371/journal.pgen.1002780

**Published:** 2012-06-21

**Authors:** Dror Sagi, Stuart K. Kim

**Affiliations:** Departments of Genetics and Developmental Biology, Stanford University Medical Center, Stanford, California, United States of America; Massachusetts General Hospital and Harvard Medical School, United States of America

## Abstract

We have taken an engineering approach to extending the lifespan of *Caenorhabditis elegans*. Aging stands out as a complex trait, because events that occur in old animals are not under strong natural selection. As a result, lifespan can be lengthened rationally using bioengineering to modulate gene expression or to add exogenous components. Here, we engineered longer lifespan by expressing genes from zebrafish encoding molecular functions not normally present in worms. Additionally, we extended lifespan by increasing the activity of four endogenous worm aging pathways. Next, we used a modular approach to extend lifespan by combining components. Finally, we used cell- and worm-based assays to analyze changes in cell physiology and as a rapid means to evaluate whether multi-component transgenic lines were likely to have extended longevity. Using engineering to add novel functions and to tune endogenous functions provides a new framework for lifespan extension that goes beyond the constraints of the worm genome.

## Author Summary

We used bioengineering to extend the lifespan of *C. elegans* by expressing genes acting in critical aging pathways. We overexpressed five genes that act in endogenous worm aging pathways, as well as two genes from zebrafish encoding molecular functions not normally present in worms. For example, we used zebrafish genes to alter mitochondrial function and innate immunity in ways not normally available to *C. elegans* and extended worm lifespan by ∼40%. Next, we used a modular approach to extend lifespan by 130% by combining up to four components in the same strain. These results provide a platform to build worms having progressively longer lifespans. This project is conceptually similar to using engineering to increase the useful lifespan of a primitive machine (1931 Model T) using both parts from the model T as well as parts from a more advanced machine (2012 Toyota Corolla). Our results open the door to use engineering to go beyond the constraints of the *C. elegans* genome to extend its lifespan by adding non-native components.

## Introduction

Recent advances in genome technology and systems biology have made it possible to use engineering approaches to create new biological systems. Examples include the construction of a synthetic genetic oscillator in bacteria [1], engineering quorum sensing (the ability to respond to population density) in yeast by integrating signaling components from the plant *A. thaliana* [2], and creating an artificial bacterial cell using a genome consisting only of chemically-synthesized DNA [3]. Here, we expand bioengineering to a complex phenotype, longevity, in a multicellular animal, *C. elegans*.

Despite being extremely complex, aging has at least three features that make it an attractive trait to improve by engineering. First, many pathways are involved in aging, such as stress response, repair of oxidative damage, protein quality control, developmental drift and innate immune response to pathogens [4]–[6]. The diverse nature of these aging pathways allows multiple avenues to engineer changes that may extend lifespan. Second, there is a great diversity in lifespan between different species, from two weeks for *C. elegans*, to 80 years for humans, to over 200 years for whales or clams [6]–[9]. This observation shows the remarkable dynamic range of over a thousand fold in lifespan encoded by different genomes. Third, most animals in the wild (including *C. elegans*) die from predation and disease rather than old age [10]–[11]. Thus, aging is not under the force of natural selection and represents the system-wide degeneration of processes due to evolutionary neglect. As a result, an engineering approach to slow aging seems more feasible than engineering improvements in other biological processes (e.g. development) because it may be easier to repair damaged processes in old animals rather than to improve highly-functional pathways in young animals.

We chose to use *C. elegans* because it has a short lifespan of two weeks and a strong genetic toolkit making it a good platform for engineering longer lifespan. We first used a variety of approaches to identify genes with well-characterized roles in critical aging pathways that can be used as components to extend lifespan in transgenic worms. In particular, we were able to extend lifespan by expressing genes from zebrafish with cellular functions that are not normally found in worms. Having created a list of components that each extends lifespan singly, we then used a modular approach to increase lifespan by increments. We generated transgenic worms that contain an increasing number of aging components, and showed that there was a corresponding increase in lifespan.

The framework and goal of our engineering approach to aging are fundamentally different from those in a study of the biology of aging. The main goal of our approach is to add components in order to extend the worm lifespan without a direct need to understand the mechanisms underlying this lifespan extension. For example, our modular approach aims to combine lifespan-extending components without aiming to determine whether these components act in the same or in different pathways. Additionally, in our engineering approach, we are not constrained to genes or pathways derived only from the worm genome. Rather, we can use novel molecular functions derived from long-lived organisms in order to extend worm lifespan.

## Results

### Four approaches to identifying components that individually extend lifespan

Our goal is to use an engineering approach to generate *C. elegans* strains that are long-lived but that develop normally, are fertile, and are generally healthy. We began by accumulating a set of genes that individually extend lifespan. The first and easiest way to obtain an aging component is to select genes that have already been shown to extend lifespan when overexpressed; we generated expression vectors for four such genes (*hsf-1*, activated *aakg-2*, *sod-1*, *daf-16*) [12]–[15]. Transgenic worms were generated by microinjection of the gene of interest, a co-transformation marker (*unc-119(+)*) and an aging biomarker (*sod-3::mCherry*). We compared the lifespan for each of the transgenic strains to the lifespan from a control transgenic strain containing *unc-119(+)* and *sod-3::mCherry* alone (see Table S1 for complete list of components). In most cases, we generated two separate transgenic strains and measured their lifespan to verify reproducibility.

**Figure 1:**
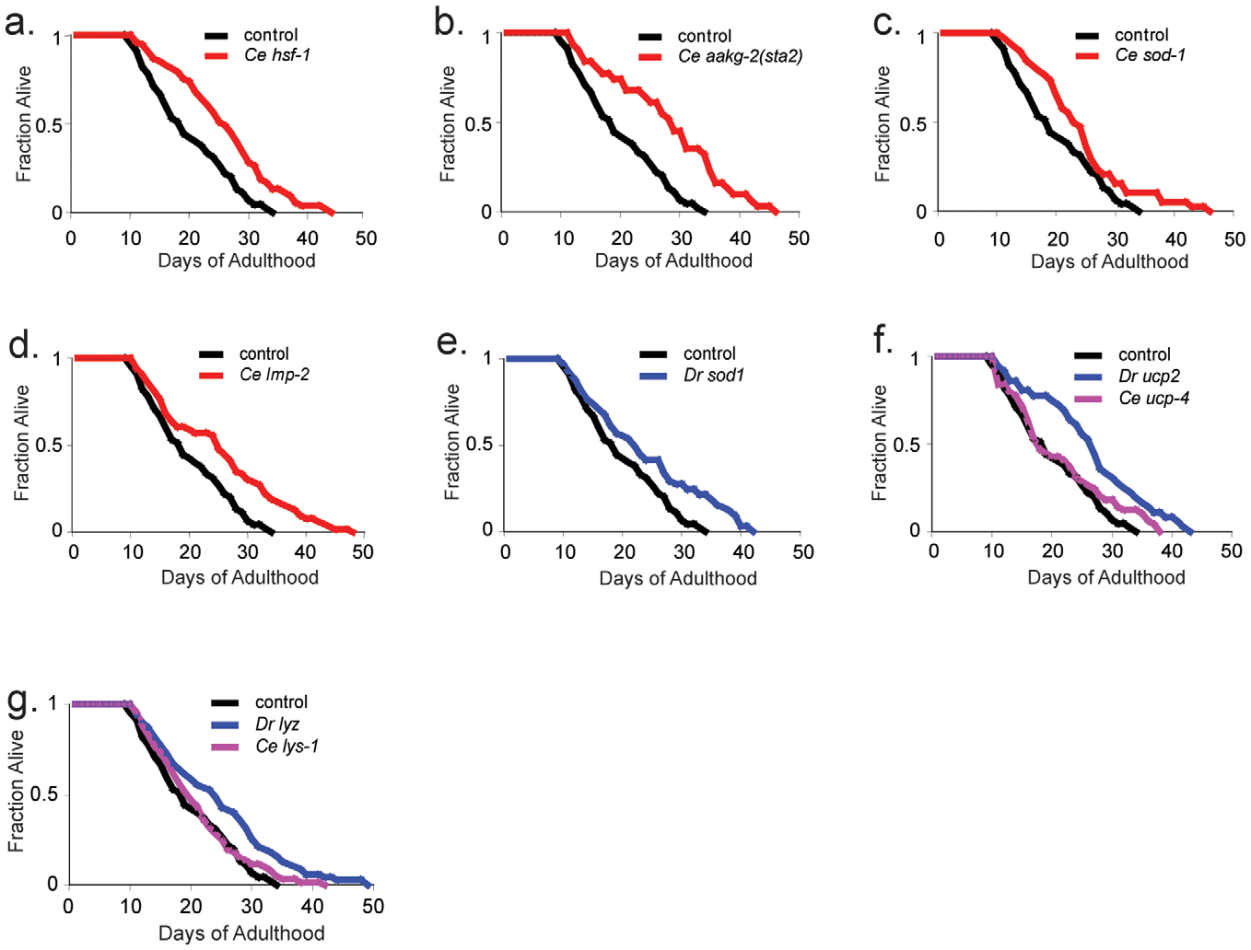
Seven aging components that can extend lifespan. a. Worm *hsf-1*. x-axis shows lifespan in days of adulthood. y-axis shows fraction of worms alive. Control refers to transgenic worms expressing *unc-119(+); sod-3::mCherry*. Worms overexpressing *C. elegans hsf-1* live 35% longer than controls (p<0.01, log-rank). b. *C. elegans* expressing *aakg-2(sta2)* live 45% longer than controls (p<0.01, log-rank). c. Worms overexpressing *C. elegans sod-1* live 25% longer than the control strain (p<0.01, log rank). d. Worms overexpressing *C. elegans lmp-2* live 34% longer than controls (p<0.01, log-rank). e. Expression of zebrafish *sod1* results in 25% lifespan extension relative to control strain (p<0.01, log rank). f. Expression of zebrafish *ucp2* results in 40% lifespan extension relative to control strain (p<0.01, log rank). Overexpression of *C. elegans ucp-4* does not show longer lifespan (p>0.05, log-rank). g. Worms expressing zebrafish *lyz* live 30% longer than control (p<0.01, log rank), whereas expression of *C. elegans lys-1* does not result in longer lifespan (p>0.05, log rank). The median lifespan of the control is 18 days. Each experiment was repeated at least three times and representative data from one experiment is shown. Additional data are listed in Table S3.

Three of the genes (*hsf-1*, activated *aakg-2*, *sod-1*) showed extended lifespan. *hsf-1* encodes heat shock transcription factor that induces expression of many stress-resistance genes that can extend lifespan [16]. *aakg-2* encodes the gamma subunit of AMP-activated protein kinase, a regulatory signaling molecule that responds to low ATP/AMP ratios and plays a key role in the stress response [17]. *sod-1* encodes cytosolic superoxide dismutase that catalyzes the dismutation of superoxide radicals (O_2_^−^) into hydrogen peroxide [18], which could reduce damage accumulation and extend lifespan. Consistent with previous results [12]–[14], we found that overexpression of *hsf-1*, activated *aakg-2* and *sod-1* extended lifespan by ∼30%, ∼45%, and 25%, respectively (Figure 1a–1c, Table 1, Table S3).

**Table 1:** Genes that extend lifespan.

Upstream region	Gene name	N^a^	Lifespan increase (%)^e^	Role
*hsf-1*	*Ce hsf-1* b	2	37±630±5	Regulates stress response
*aakg-2*	*Ce aakg-2(sta2)* c	2	48±645±10	Kinase, senses ATP:AMP ratios
*sod-1*	*Ce sod-1* d	1	25±7	Reduces O_2_^−^
*lmp-2*	*Ce lmp-2*	2	33±435	Receptor, Induces chaperone-mediated autophagy
*sod-1*	*Dr sod1*	2	25±525±4	Reduces O_2_^−^
*ucp-4*	*Dr ucp2*	2	44±335±4	Uncoupling protein, reduces proton gradient
*lys-1*	*Dr lyz*	1	28±5	Lyses bacterial cell wall

a Number of lines;

b see also [12];

c see also [13];

d see also [14];

e median percentage increase in lifespan ± SD from three independent assays (p<0.01 determined by log-rank statistics); at least 80 worms were scored in each lifespan assay. Additional data are shown in Table S3.

The second way to obtain an aging component is a candidate gene approach using *C. elegans* genes that act in known aging pathways. One such aging pathway is proteostasis, which counteracts damage accumulation to proteins by removing old, damaged proteins [19]–[20]. Increasing the rate of protein turnover should lower accumulation of damaged proteins and may extend lifespan [21]. We overexpressed a gene involved in chaperone-mediated autophagy (*lmp-2*) [22] and a gene involved in proteostasis (*uba-1*). We found that that *lmp-2* but not *uba-1* resulted in extended lifespan compared to a control strain (Figure 1d, Table 1, Table S3). *lmp-2* is the ortholog of mammalian *lamp2A*, which encodes the lysosome-associated membrane protein type 2A receptor involved in chaperone-mediated autophagy that is responsible for the degradation of approximately 30% of cytosolic proteins in conditions of stress [23]. Overexpression of *lamp2A* in old mice results in lower intracellular accumulation of damaged proteins and improved organ function [22].

The third approach was expressing orthologous genes from either the zebrafish or the human genome that act in known aging pathways. We selected genes from zebrafish and humans as they have much longer lifespans than worms (4 years or 80 years vs 2 weeks, respectively) [6], [8], [24]. We expected that vertebrate genes from aging pathways may be more efficient at delaying aging than orthologous genes from worms. Furthermore, zebrafish live at a similar range of temperatures as *C. elegans* and therefore zebrafish proteins should be capable of functioning at the ambient temperature used to grow worms (20°C).

We selected four zebrafish and one human gene that are orthologous to *C. elegans* genes that act in known aging pathways: *D. rerio sod1, D. rerio msra, D. rerio foxo3A*, *D. rerio psmb1* and human *aldh2*. We used upstream regions from *C. elegans* genes that were homologous to the zebrafish gene to drive expression of zebrafish cDNAs. For each construct, we generated transgenic worms and measured their lifespan under normal growth conditions. Of the five genes tested, only expression of *D. rerio sod1* in transgenic worms resulted in longer lifespan than a control strain (Figure 1e, Table 1, Table S3). *D. rerio sod1* encodes superoxide dismutase and is the ortholog of *C. elegans sod-1*. *D. rerio sod1* and *C. elegans sod-1* extended lifespan to a similar extent.

The fourth approach to find an aging component was to select zebrafish genes with functions that are absent from the worm genome, and test whether adding them to worms can have a beneficial effect. One such function is mitochondrial uncoupling, which allows protons to leak into mitochondria without producing ATP [25]. According to the uncoupling to survive hypothesis, mitochondrial proton leakage may be beneficial because reduction of the proton motive force should reduce production of reactive oxygen species and thereby reduce damage accumulation during aging [25]. We chose to introduce mitochondrial uncoupling activity into worms using the zebrafish *ucp2* gene, which encodes one of the mitochondrial uncoupling proteins. A similar experiment to add human *ucp2* to *Drosophila* has been done previously, although it is not clear whether *Drosophila* has endogenous uncoupling activity and thus it is unclear if this previous experiment involves adding a new function to *Drosophila* [26].

The evolutionary tree for *ucp* genes shows that *ucp-4* is the most ancient, contained in the genomes of all animals (Figure S1a). Worms contain only a single related gene (*ucp-4*) that encodes a protein that does not have mitochondrial uncoupling activity but rather is a transporter for succinate [27]–[28].

We generated transgenic worms that express zebrafish *ucp2* from the worm *ucp-4* promoter. As a control, we also generated worms that overexpress worm *ucp-4*. We found that expression of zebrafish *ucp2* extended the median lifespan of worms by about 40% (Figure 1f, Table 1, Table S3). In contrast, overexpression of worm *ucp-4* did not extend lifespan in two independent transgenic strains (Figure 1f, Table S2).

Another example of new functionality added to the worm is the addition of vertebrate lysozyme activity. Worm lifespan is limited by mild pathogenic effects from *E. coli*, which is used as a standard food source [29]–[30]. All lysozymes have bacterial cell wall hydrolase activity that degrades peptidoglycans and thus are key players of the innate immune defense system providing protection against bacterial pathogens [31]. There are ten lysozyme genes in *C. elegans*, all belonging to a clade shared with microbes such as *D. discoideum* and *E. histolytica* (Figure S1b). Vertebrates contain a large number of lysozyme genes, including a second clade that is derived solely from metazoans. Lysozyme genes from this clade contain a distinct anti-bacterial activity besides cell wall hydolase activity, which involves direct interaction of lysozyme with the bacterial cell membrane resulting in membrane leakage [31].

We generated transgenic worms expressing a zebrafish lysozyme gene from the second clade (*lyz*). We found that zebrafish *lyz* extended the median lifespan of worms by about 30% (Figure 1g, Table 1, Table S3). In contrast to *D. rerio lyz*, overexpression of worm *lys-1* did not extend lifespan in two independent transgenic strains (Figure 1g, Table S2), consistent with previously published results [32].

### Effects of aging components on worm and cell physiology

We used various worm- and cell-based assays to validate that the aging components were expressed and to determine what changes in cell physiology and stress were induced. One reason for this is to provide evidence that the aging components extended lifespan by the expected mechanism. Since we tested each of the seven aging components with each of the assays, another reason is to also determine whether the aging component induced unexpected changes in cell physiology, which might indicate indirect activation of secondary aging pathways. A third reason is that the cellular assays could be used as a rapid and practical means to identify transgenic worms that are likely to have extended lifespan.

To examine expression of the transgenes, we performed RT-PCR experiments using RNA extracted from fourth larval stage hermaphrodites. We found that the three vertebrate genes were expressed and that the four worm aging genes were over-expressed 5–18 fold in the transgenic strains compared to the endogenous gene in the control strain (Table S4).

**Figure 2:**
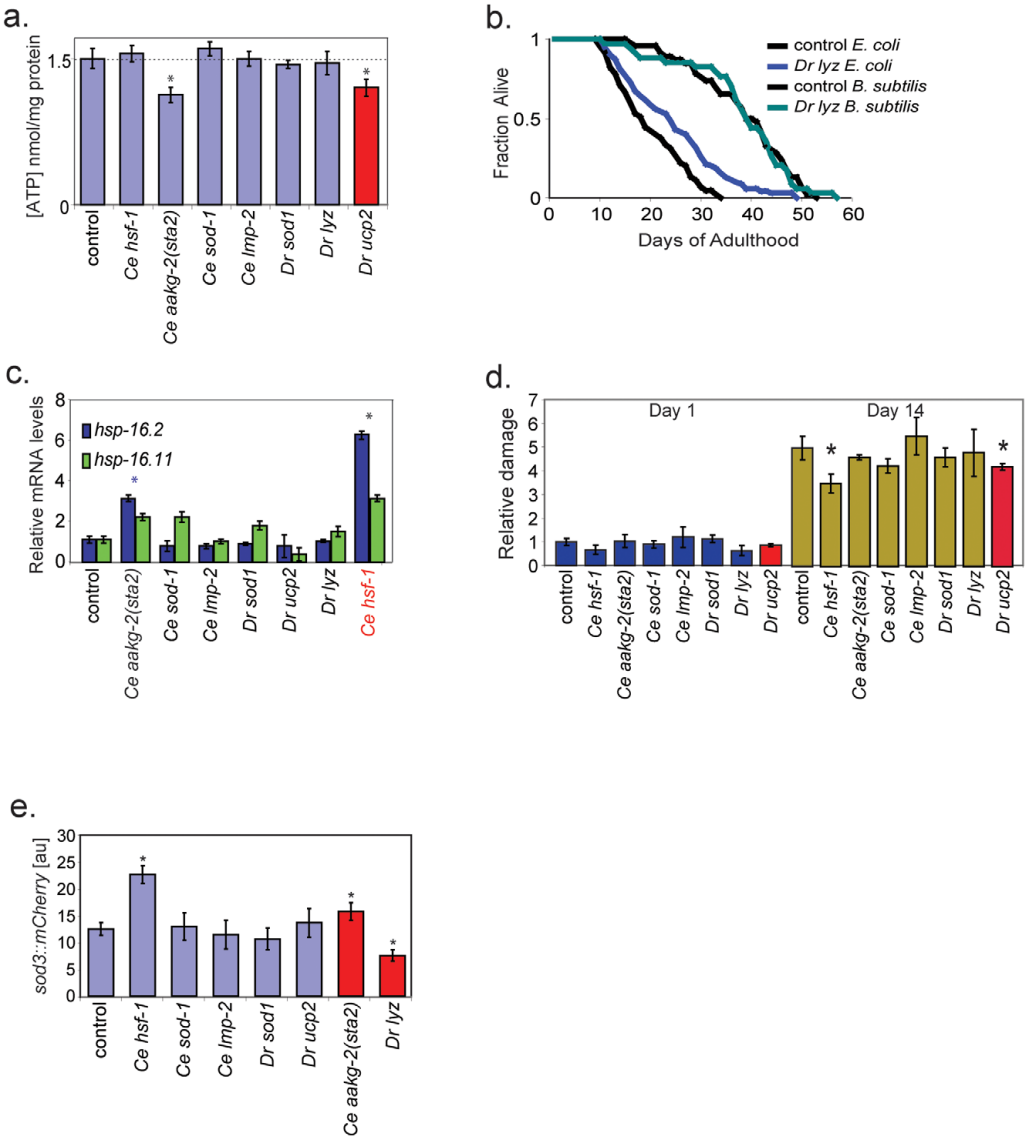
Assays showing functional activity of the aging genes. a. Levels of total ATP in extracts from L4 stage hermaphrodites. ATP levels were measured using a luciferase bioluminescent assay. y-axis shows ATP levels (nmoles per mg of protein). * indicates p<0.01, t-test. Error bars are SEM of three independent experiments. Red bar indicates transgenic line expected to show decreased level of ATP. b. *B. subtilis* does not extend the lifespan of worms expressing *D. rerio lyz*. Worms expressing *D. rerio lyz* live longer than control strains when grown on *E. coli* (p<0.01, log-rank) but not when they are grown on *B. subtilis* (p>0.05, log rank). Shown are representative data from one of six experiments. At least 80 worms were score in each experiment. Additional data are in Table S4. c. Induction of *hsp-16.2* and *hsp-16.11* in worms expressing either *C. elegans hsf-1* or *C. elegans aakg-2(sta2)*. y-axis shows normalized RNA levels of either *hsp-16.11* or *hsp-16.2* relative to control, measured by qPCR. Error bars are SEM of three independent experiments. Each bar represents transgenic worms expressing the corresponding gene. * indicates p<0.01 (t-test) for both *hsps* . Red text indicates transgenic line expected to show increased expression of *hsp-16.2* and *hsp-16.11*. d. Oxidative damage to proteins in long-lived transgenic worms. Oxidative damage levels in worm extracts increase about five fold from young adult (day 1) to old adult (day 14). At 14 days of adulthood, transgenic worms expressing *D. rerio ucp2* or *C. elegans hsf-1* have less damage (*, p<0.05, t-test). Oxidative damage to proteins was measured by the Oxiblot method, and is expressed relative to oxidative damage in a control strain in arbitrary units. Each bar represents transgenic worms expressing the corresponding gene. Error bars are SEM of three independent experiments. Red bars indicate transgenic lines expected to show decreased levels of oxidative protein damage. e. Expression of *sod-3::mCherry*. Worms expressing *C. elegans hsf-1* or *C. elegans aakg-2(sta2)* show higher expression (p<0.01, t-test) and worms expressing *D. rerio lyz* show lower expression (p<0.01, t-test) of *sod-3::mCherry*. y-axis show fluorescence in arbitrary units. Error bars are SEM of three independent experiments. Each bar represents transgenic worms expressing the corresponding gene. Red bar indicates transgenic line expected to show increased expression of *sod-3::mCherry*. *Ce* – *C. elegans*, *Dr* – *D. rerio*.

ATP production by the mitochondria is directly related to the production of reactive oxygen species and damage accumulation. Furthermore, ATP levels is thought to be associated with dietary restriction and the subsequent induction of protective pathways [33]–[34]. We measured ATP levels in extracts from fourth larval stage worms for each of the transgenic worms and found that worms expressing *ucp2* or *aakg-2(sta2)* had lower overall levels of ATP compared to controls (Figure 2a). Uncoupling protein would be expected to lower ATP levels by lowering the proton gradient in mitochondria, and thus lowering ATP production. Our results are consistent with previous experiments showing that vertebrate *ucp2* genes have mitochondrial uncoupling activity when expressed in yeast and in flies [26], [35]–[36]. Activation of AMPK is thought to increase catabolic pathways that generate ATP while decreasing ATP-consuming processes [17]. Thus, *aakg-2(sta2)* transgenic worms were expected to have increased ATP levels, opposite to the observed result.

Lysozymes are anti-bacterial enzymes that could extend lifespan by combating bacterial pathogenicity. If lysozyme acts by combating mild pathogenicity stemming from *E. coli*, then it should not be able to extend lifespan when worms are grown on non-pathogenic *B. subtilis*. We determined the lifespan of control and *lyz* transgenic worms when grown on *B. subtilis*, and found no difference (Figure 2b, Table S5). This result strongly indicates that the mechanism of lifespan extension by zebrafish lysozyme involves combating mild pathogenicity from *E. coli*.

Mild stress can extend lifespan by inducing protective pathways, a phenomenon referred to as hormesis. We tested for induction of the stress-responsive genes *hsp-16.2* and *hsp-16.11* using RT-PCR. We found that *hsf-1* and *aakg-2(sta2)* transgenic worms showed higher expression of *hsp-16.2* and *hsp-16.11* than controls (Figure 2c). *hsf-1* but not *aakg-2(sta2)* was expected to induce expression of stress response genes.

Next, we examined resistance to oxidative damage, which accumulates with age. One way to measure susceptibility to oxidative damage is to measure resistance to oxidative stress from paraquat, a chemical that generates superoxide ions. We found that *C. elegans sod-1*, zebrafish *sod1, aakg-2(sta1)* and *hsf-1* conferred resistance to paraquat (Table 2). *C. elegans sod-1* and zebrafish *sod1* encode superoxide dismutase, an enzyme that reduces levels of oxygen free radicals that could directly counteract the effects of paraquat. *aakg-2(sta2)* and *hsf-1* were not expected to affect oxidative damage directly.

**Table 2:** Resistance to paraquat.

Gene	200 mM paraquat^a^	50 mM paraquat^a^
Control	8.75	32
*Ce hsf-1*	13*	45*
*Ce aakg-2(sta2)*	12.5*	44.3*
*Ce sod-1*	11.6*	42.2*
*Ce lmp-2*	8	32.5
*Dr sod-1*	11.3*	40*
*Dr ucp2*	8	25.6*
*Dr lyz*	8.6	32.5
Dual-1[*Ce aakg-2(sta2); Dr ucp2*]	14.22*	
Dual-2[*Ce hsf-1; Dr lyz*]	13.77*	
Triple-1[*Ce aakg-2(sta2); Dr ucp2; Dr lyz*]	13.5*	
Triple-2[*Ce hsf-1; Dr lyz; Ce aakg-2(sta2)*]	13.33*	
Quadruple[*Ce hsf-1; Dr lyz; Ce aakg-2(sta2); Dr ucp2*]	17.6*	

a Median lifespan in hours (N = 150 worms).

*: p<0.05, determined by log-rank statistics.

Another way to examine oxidative damage in worms is to directly detect oxidized residues in proteins from a whole worm extract in a Western blotting assay. In old worms, *ucp2* and *hsf-1* worms showed lower levels of oxidative damage compared to controls (Figure 2d). *ucp2* could decrease levels of oxidative damage by decreasing the proton motive force in mitochondria and reducing production of reactive oxygen species. Reduced levels of oxidative damage in *hsf-1* transgenic worms was not anticipated. *C. elegans sod-1* and *D. rerio sod1* might be expected to reduce oxidative damage by reducing levels of reactive oxygen species, but neither showed an effect in this assay. Tallying the results from both assays for oxidative damage, we found expected evidence for reduced oxidative damage in three strains (*ucp2* and *C. elegans sod-1, D. rerio sod1*) as well as unanticipated results for two strains (*hsf-1* and *aakg-2(sta2)*).

We next examined activation of the FOXO transcription factor DAF-16, which is a key regulator of aging [6]. Activation of DAF-16 can be measured by expression of a *sod-3* reporter gene, which is one of its downstream targets [37]. We compared the level of expression of a *sod-3::mCherry* reporter in each of the seven long-lived worms to control worms in middle-aged hermaphrodites. We observed that *aakg-2(sta2)* and *hsf-1* transgenic worms showed increased expression of *sod-3::mCherry* whereas zebrafish *lyz* showed decreased expression (Figure 2e). *aakg-2* encodes a kinase that phosphorylates DAF-16, and would be expected to induce *sod-3* expression [13], [38]. For zebrafish *lyz*, one possibility is that lysozyme could reduce pathogenicity from *E. coli* used as food. Mild pathogenicity is known to activate DAF-16 and induce expression of the downstream target *sod-3*.

**Figure 3:**
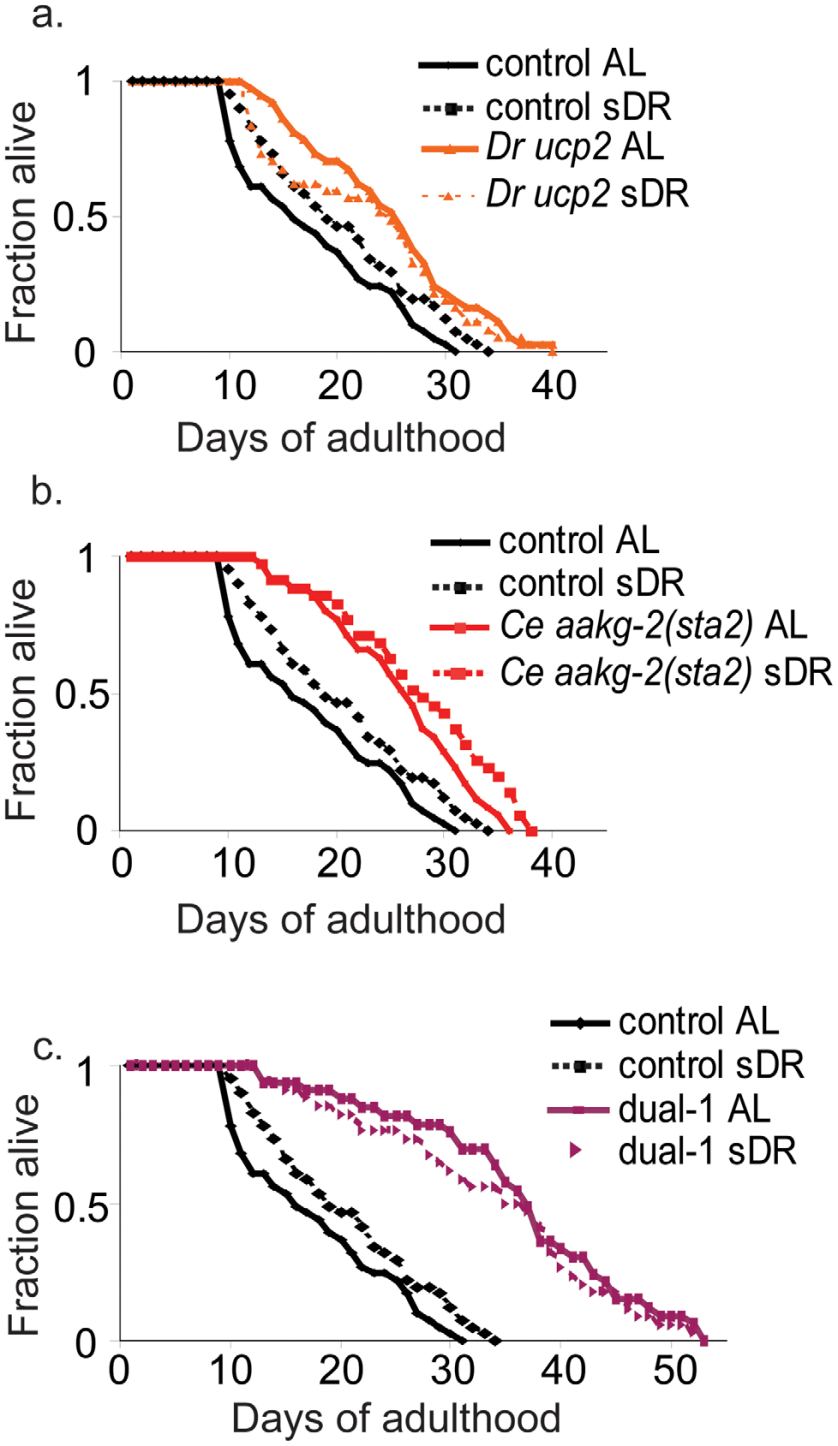
Dietary restriction does not increase lifespan of the *D. rerio ucp2*, *C. elegans aakg-2(sta2)*, or Dual-1 strains. (a) *D. rerio ucp2*, (b) *C. elegans aakg-2(sta2)*, and (c) Dual-1 strains. y-axis shows fraction of worms alive, x-axis shows lifespan of synchronized worms starting from adulthood. *Dr* – *D. rerio*. Control refers to an *unc-119(+); sod-3::mCherry* strain. sDR indicates feeding on plates with 10^8^ CFU. AL indicates feeding on plates seeded with 10^11^ CFU. Control worms on sDR live 18% longer than control worms on AL (p<0.01, log rank). A second independent assay showed similar results to those shown (Table S5).

Lastly, lower ATP levels in *ucp2* transgenic worms might extend lifespan using mechanisms shared by dietary restriction. If so, then worms that receive both dietary restriction and *ucp2* might not live longer than worms receiving either condition alone. We found that dietary restriction alone extended median lifespan 18%, *ucp2* alone extended lifespan 40% and that dietary restriction of *ucp2* worms extended lifespan 40% compared to controls (Figure 3a, Table S6). Thus, dietary restriction did not further extend the lifespan of *ucp2* worms that were fed normally.

Table 3 provides a summary of the results from the seven cell- and worm-based assays using the seven transgenic lines expressing aging components. Except for *lmp-2*, we obtained either direct or indirect evidence that each of the components was expressed and acting as expected. Furthermore, we also obtained evidence that some aging components produced changes in cell pathways that were indirect, providing evidence for cross-talk between different aging pathways in *C. elegans*. For example, the *aakg-2(sta2)* strain also shows induction of the two *hsp* protein chaperones that function in a protective stress pathway.

**Table 3:** Summary of cell assays.

	*hsf-1* a	*aakg-2(sta2)* a	*sod-1* a	*lmp-2* a	*sod1* b	*ucp2* b	*lyz* b
**ATP levels**	**same**	**low**	**same**	**same**	**same**	lower	**same**
Extends lifespan on *B. subtilis*	ND	no effect	ND	ND	ND	ND	**No effect**
RNA levels of *hsp-16.2* and *hsp-16.11*	**higher**	higher	same	same	same	same	same
Paraquat resistance	higher	higher	**higher**	same	**higher**	same	same
Levels of carbonyls	lower	same	same	same	same	**lower**	same
*sod-3::mCherry* levels	higher	**higher**	same	same	same	same	**lower**
Lifespan with dietary restriction	ND	**same**	ND	ND	ND	**same**	ND

a *C. elegans*;

b *Danio rerio*; **Bold** indicates expected changes. ND not determined.

### Extension of lifespan does not necessarily affect brood size

According to the disposable soma theory, evolution of organisms in the wild requires a balance between allocation of metabolic resources for somatic maintenance or reproduction [10]. We tested whether there was a reduction in brood size in our engineered strains. Five transgenic strains with long lifespan had similar brood size and two showed a decrease in fertility compared to the control strains (Figure S2). These results show that the aging components can extend lifespan without reducing fertility.

### Using a modular approach to progressively increase lifespan

The seven aging components are individually capable of extending lifespan 25–50%. Because aging is a complex phenomenon affected by many pathways, our strategy to extend lifespan further was to use a modular approach by combining different aging components in a single transgenic strain to progressively extend lifespan. Additionally, we needed to develop a scheme to rapidly test whether or not combining genes in a new transgenic strain has a beneficial effect. This is because lifespan analysis requires four weeks for normal worms, and becomes even more tedious as lifespan increases. Our approach was to first use the cell- and worm-based assays described above to rapidly test whether worms expressing multiple aging components show a beneficial effect. Results showing that a multi-component strain shows protective changes in several pathways or stronger effects in a single aging pathway compared to single-components lines would be encouraging that it will live a long time.

We started by generating two transgenic worm strains that each contain two components; one combination (dual-1) contains *aakg-2(sta2)* and zebrafish *ucp2* and the other combination (dual-2) contains *hsf-1* and zebrafish *lyz* (Table 4). These four components include two zebrafish genes that add new functionality to the worm (*ucp2* and *lyz*) and two *C. elegans* genes that showed the largest increase in lifespan (*aakg-2(sta2)* and *hsf-1*). We used qRT-PCR to show that the components in the dual-module worms were expressed at levels equivalent to those from the single-module worms (Table S4).

**Table 4:** Lifespan extension is modular.

Gene	N^a^	Lifespan increase (%)^b^	p-value	control
*Ce aakg-2(sta2)*	2	48±645±10	4×10^−5^4×10^−5^	*unc-119(+)* *unc-119(+)*
*Dr ucp2*	2	44±335±4	2×10^−3^7×10^−3^	*unc-119(+)* *unc-119(+)*
*Ce hsf-1*	2	37±630±5	5×10^−3^9×10^−3^	*unc-119(+)* *unc-119(+)*
*Dr lyz*	1	28±5	10^−2^	*unc-119(+)*
Dual-1[*Ce aakg-2(sta2); Dr ucp2*]	2	80±1084±10	8×10^−3^5×10^−3^	*Ce aakg-2(sta2)* *Ce aakg-2(sta2)*
Dual-2[*Ce hsf-1; Dr lyz*]	2	60±957±5	2×10^−4^4×10^−4^	*Ce hsf-1* *Ce hsf-1*
Triple-1[*Ce aakg-2(sta2); Dr ucp2; Dr lyz*]	2	105±1097±8	2×10^−4^4×10^−3^	dual-1dual-1
Triple-2[*Ce hsf-1; Dr lyz; Ce aakg-2(sta2)*]	2	92±1084±12	6×10^−5^2×10^−4^	dual-2dual-2
Quadruple[*Ce hsf-1; Dr lyz; Ce aakg-2(sta2); Dr ucp2*]	2	135±14124±17	5×10^−4^	triple1

a Number of lines;

b median percentage increase in lifespan ± SD (p<0.01 determined by log-rank statistics) from three independent assays. At least 80 animals were scored for each lifespan experiment; Additional data are shown in Table S7.

**Figure 4:**
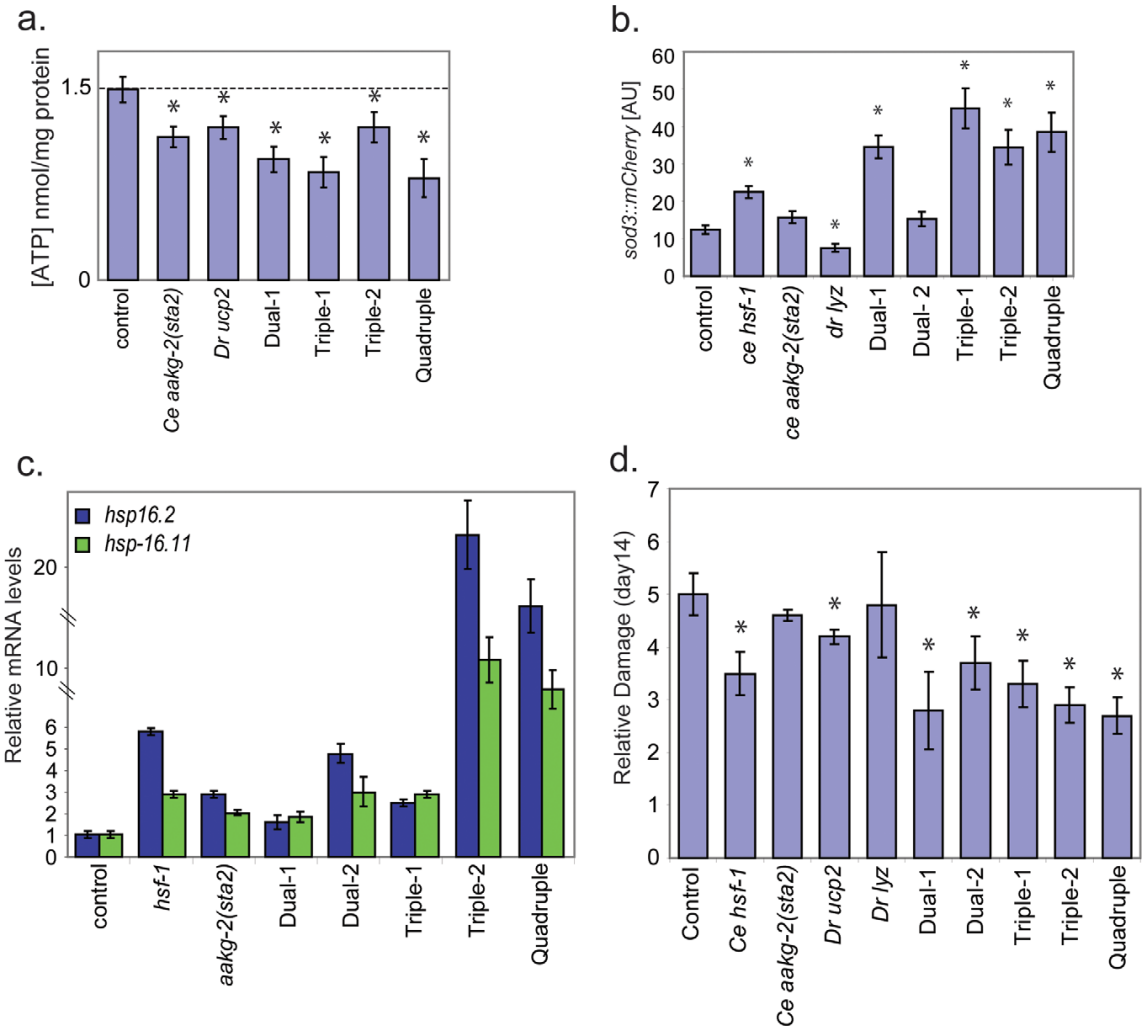
Engineering longer lifespan is modular. a. ATP levels for double-, triple-and quadruple-expressing transgenic worms. y-axis shows ATP levels (nmoles) normalized to mg of protein (mean and SEM of three independent experiments). * indicates p<0.01, t-test. b. Fluorescence levels of *sod-3::mCherry* reporter. y-axis shows fluorescence in arbitrary units from quantitative measurement of 10 worm images using ImageJ. Error bars are SEM of three independent experiments. * indicates p<0.01, t-test. c. Induction of expression of *hsp* genes. y-axis shows normalized RNA levels measured by qPCR of either *hsp-16.11* or *hsp-16.2* relative to a control strain (*unc-119(ed3); gaEx[unc-119(+); sod-3::mCherry]*). Error bars are SEM of three independent experiments. In all cases p<0.01, t-test. d. Oxidative damage to proteins. Oxidative damage levels in old worms (day 14). At 14 days of adulthood, all dual-, triple– and quadruple-expressing transgenic worms have less damage than control worms (*, p<0.05, t-test). Oxidative damage to proteins was measured by the Oxiblot method, and is expressed relative to oxidative damage in a control strain in arbitrary units. Each bar represents transgenic worms expressing the corresponding gene. Error bars are SEM of three independent experiments.

One reason for choosing the two components in dual-1 was that six assays were affected by either *aakg-2(sta2)* or *ucp2* single-expressing worms, suggesting that this combination might be able to affect a large number of aging pathways. We analyzed dual-1 worms with all six assays to examine changes in cell physiology resulting from expression of the two aging components. For four assays (ATP level, paraquat resistance, oxidative damage and *sod-3* expression), the change relative to controls seen for the dual-1 strain was greater than the changes seen with either the *aakg-2(sta2)* or *ucp2* single strains (Figure 4a, 4b, 4d; Table 2). For the dietary restriction assay, the results with dual-1 were similar to *aakg-2(sta2)* and *ucp2* single-module worms; specifically, the lifespan of dual-1 was not further extended by dietary restriction (Figure 3a–3c). For induction of the *hsp* genes, the dual-1 strain showed a smaller change than the *aakg-2(sta2)* strain by itself (Figure 4c). Thus, the dual-1 strain showed changes in a greater number of assays than in either of the single-expressing lines, and oftentimes the changes were larger in magnitude.

The dual-2 strain contains components (*hsf-1* and *D. rerio lyz*) that showed changes in a total of five cell assays when expressed singly (Table 3). We tested dual-2 in four of the cell assays (paraquat resistance, *hsp* induction, oxidative damage and *sod-3* expression), and saw changes in the first three but not *sod-3* expression with respect to control worms (Figure 4a–4d, Table 2). Paraquat resistance was greater in dual-2 than in each of the two single lines, but changes in *hsp* induction and oxidative damage relative to control worms was less or equivalent to the changes found in the *hsf-1* and *D. rerio lyz* single-module lines (Figure 4c, 4d; Table 2). For *sod-3*, *hsf-1* increases but *D. rerio lyz* decreases its expression. In dual-2, *sod-3* expression is not different than in control worms suggesting that the opposite effects from these two genes cancel out (Figure 4b).

**Figure 5:**
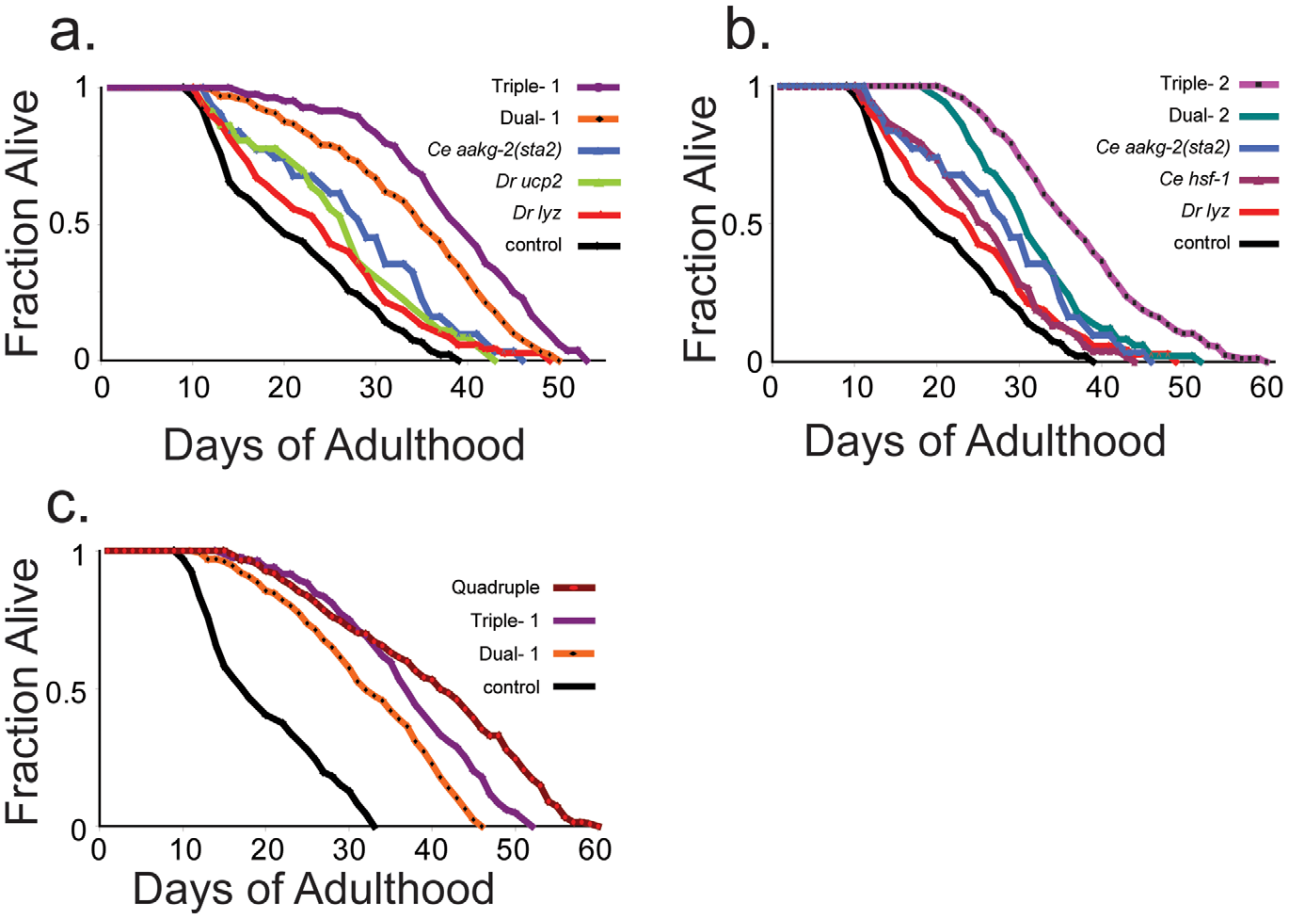
Lifespan curves of double-, triple-, and quadruple-expressing transgenic worms. (a,b,c) y-axis shows fraction of worms alive, x-axis shows days of adulthood. At least 80 worms were scored for each lifespan curve. Shown is one representative lifespan assay for each strain. Lifespan data from three independent assays are shown in Table S7.

Finally, we measured the lifespan of the dual-expressing lines, and compared them to the lifespan of the single-expressing lines and controls. Two independent dual-1 lines had an increase in median lifespan of ∼80%, compared to an increase of 35–48% from either of the single components (Figure 5a, Table 4, Table S7). Two independent dual-2 lines had an increase in median lifespan of ∼60%, compared to an increase of 28–37% from either of the single components alone (Figure 5b, Table 4, Table S7). These results show that expression of two components can have an additive effect on lifespan.

We extended the method by generating transgenic lines that each express three components. Specifically, triple-1 was generated based on dual-1 with the addition of *D. rerio lyz*, and thus contains *aakg-2(sta2)*, *D. rerio ucp2* and *D. rerio lyz*. Triple-2 was based on dual-2 with the addition of *aakg-2(sta2)*, and thus contains *hsf-1, D. rerio lyz* and *aakg-2(sta2)*. We examined expression of the components in the triple-expressing lines using qRT-PCR, and found that each of the genes in the triple-expressing lines was expressed at levels comparable to those from the corresponding single-expressing line (Table S4).

For triple-1 and triple-2, the three constituent aging components can affect each of the seven assays when expressed individually. We examined triple-1 and triple-2 using five of the cell assays (ATP level, *hsp* induction, paraquat resistance, oxidative damage and *sod-3* expression). Triple-1 and triple-2 showed changes in all five assays with respect to control worms (Figure 4a–4d, Table 2). We then measured the lifespan of the two triple-expressing lines and found that two independent triple-1 lines showed 97–105% increased lifespan and two independent triple-2 lines showed 84–92% increased lifespan (Figure 5a, 5b; Table S7).

Finally, we generated a quadruple-expressing line containing four different components: *hsf-1*, *D. rerio lyz, aakg-2(sta2)* and *D. rerio ucp2*. We measured expression of these genes in the quadruple line to determine if their expression was as high in the quadruple-expressing as in the single-expressing lines. We found that *hsf-1* was expressed at a comparable level but that *aakg-2(sta2)*, *Dr ucp2* and *Dr lyz*, were expressed at about 50% of the previous levels (Table S4). The quadruple-expressing line showed changes in all five cell assays with respect to control worms, and was the most resistant to paraquat among all strains (Table 2 and Figure 4a–4d). The lifespan of this quadruple-expressing line was increased 130% compared to control, and this result was verified in a second quadruple-expressing line (125% increased)(Figure 5c, Table S7). Taken together, our results show a monotonic increase in lifespan: single-expressing lines (28–47%), double-expressing lines (57% to 84%), triple-expressing lines (84–105%) and quadruple-expressing lines (125–130%).

Besides living for the longest time of any of the engineered strains, the quadruple also has a long health span. Quadruple worms reach the L4 larval stage in approximately 72 hours, similar to control worms. Young adult quadruple worms appear and move normally when viewed using a dissecting microscope. For control worms, the median lifespan is about 18 days at which point most of the animals still alive show limited mobility. For quadruple worms, the median lifespan is 40 days but most of the surviving animals move well, similar to 14 day-old control worms (Videos S1, S2, S3). These observations indicate that we have extended the time that quadruple worms are mobile and healthy. This is important for lifespan engineering, as one would optimally want to extend the healthy portion over the morbid time at the end of life.

## Discussion

This paper uses an engineering approach to build healthy and long-lived worms. Our approach was to choose genes from well-studied aging pathways that can be used as components to extend lifespan, and then validate that they are active using a variety of molecular and cellular assays. In this initial study, we used four approaches to find seven aging components. In the first approach, three components (*hsf-1*, *aakg-2(sta2)* and *sod-1*) were obvious choices because they were previously known to extend lifespan when overexpressed in worms [12]–[14]. Future genetic studies of aging are likely to reveal many more genes that extend lifespan when overexpressed, each time providing a new aging component.

Secondly, one component was chosen based on prediction from theory; specifically, *lmp-2* was selected because it is involved in chaperone-mediated autophagy [23]. Overexpression of this component is expected to increase protein degradation, reduce steady-state levels of protein damage, and thereby extend lifespan. In this case, not only did we generate an aging component that can be used in our study, but we were also able to validate a prediction made from theory and thus provide support for the role of protein turnover in aging.

In the third approach, we used orthologous genes from zebrafish rather than genes from *C. elegans*. We found *D. rerio sod1* and *C. elegans sod-1* extended lifespan to a similar extent. It will be interesting to continue to compare orthologous genes from *D. rerio* and *C. elegans* in order to determine whether there may be a systematic advantage to selecting genes from a longer-lived species.

Lastly, we showed that we can extend lifespan by expressing new functions in *C. elegans*. The first function is mitochondrial uncoupling activity, which is absent from *C. elegans* [28]. Previous work has shown that human *ucp2* extends the lifespan of *D. melanogaster*, although it is not clear whether this involves adding a new activity to flies because it is not known whether flies have an endogenous mitochondrial uncoupling activity [26]. The second novel function is vertebrate lysozyme, which has an additional anti-bacterial function not found in *C. elegans* lysozymes [31]. We found that worms expressing either uncoupling protein or lysozyme from *D. rerio* have a longer lifespan than control worms.

Each of the aging components can extend lifespan about 30–50% by themselves. To extend lifespan beyond this amount, we combined four different aging components in the same line and extended lifespan to 130%. As the number of components increases beyond four, lifespan assays will become more time-consuming and less practical. For practical purposes, we showed that we can rapidly use cell- and worm-based assays to assess whether a multi-component strain is a good candidate for extended longevity, before having to perform the lifespan assay itself. There was generally good agreement between the results from the cell assays and lifespan; e.g, high levels of *sod-3* expression correlated well with extended lifespan in the multi-component strains. It will be interesting to determine how much further one can extend lifespan by adding additional components. Previous studies have already shown that *daf-2* mutant worms that lack a germline have a five-fold increase in longevity [39]. Future engineering efforts may also be able to achieve extreme longevity.

Although data from our cell assays indicate that a certain aging pathway may be active, it is difficult to formally conclude that the observed activity is the cause for longer lifespan. This is because any of the aging components may have an unknown second activity. For instance, our results show that expression of superoxide dismutase results in paraquat resistance, consistent with a reduction in oxidative damage. However, recent evidence suggests that this enzyme extends lifespan not through an oxidative damage pathway but by another undefined mechanism [40]. Whether or not the precise mechanism is reduction of oxidative damage, superoxide dismutase does indeed extend lifespan and serves our purposes as an aging component to engineer longer-lived worms.

This work provides a proof of principle that one can engineer longer lifespan in *C. elegans* by adding new components. New technologies in DNA construction, increased knowledge of aging pathways, and improved methods to fine-tuning gene expression will add powerful tools to engineering lifespan. For instance, it will soon be possible to synthesize large stretches of DNA containing many genes from any organism, worm or otherwise, in order to express multiple genes from a genetic pathway. For example, the innate immune system of vertebrates is a source of new anti-bacterial proteins that could significantly improve resistance to pathogenicity in *C. elegans* [41]. Additionally, expression of vertebrate-specific chaperones and mitochondrial proteins in worms may improve their proteostasis and energy balance pathways, respectively [42]–[43]. Adding exogenous components from vertebrates is a powerful strategy that goes beyond the natural constraints of the *C. elegans* genome to engineer worms with increased lifespan and healthspan.

## Materials and Methods

### *C. elegans* genetics

All *C. elegans* strains were maintained and handled as previously described [44]. 5-fluoro-2′-deoxyuridine (FUDR, Sigma) plates were made by supplementing NGM agar media with 30 µM of FUDR.

Genes from *C. elegans* used in this study were amplified by PCR from N2 worm genomic DNA. Generation of constructs containing zebrafish or human cDNA used worm upstream regulatory sequence as defined by the promoterome [45]. If the required promoter was not part of promoterome, all intergenic sequence upstream of the gene of interest was used. cDNA of the gene of interest was obtained from Open Biosystems. The 3′ UTR was from the intron-containing *unc-54* gene.

Transgenic strains were made by microinjecting *unc-119* worms with the gene of interest at 10 ng/µl and PD4H1 (*unc119(+); sod3::mCherry*) [46] at 80 ng/µl. To generate transgenic worms containing two or three genes, each of the genes of interest was injected at 10 ng/µl and PD4H1 at 70 ng/µl. *sod-3::mCherry* is a reporter for *daf-16* activity.

### Analysis of life span

Life span analyses were conducted on FUDR plates at 20°C as previously described [47]. At least 80 worms were used for each experiment. Age refers to days following adulthood, and p-values were calculated using the log-rank (Mantel-Cox) method. Individuals were excluded from the analysis if their gonad was extruded or if they desiccated by crawling onto the edge of the plate.

### RNA levels

Fourth larval stage worms were washed with M9 buffer and pelleted in a centrifuge. RNA was extracted by addition of 500 µl of Trizol (Invitrogen) to 50 µl of worm pellet, followed by six freeze-thaw cycles in liquid nitrogen. RNA extraction was performed according to the Trizol protocol from the manufacturer. Gene expression was determined by reverse transcription of 0.5 µg total RNA with the Superscript III kit (Invitrogen) followed by quantitative PCR analysis on a Step One Plus real time PCR machine (Applied Biosystems) with iQ SYBR green (Bio-Rad) using *act-1* RNA as a control. The experiments were conducted in triplicate. Expression level of a gene of interest relative to *act-1* was determined by calculating the difference in the number of cycles between the gene of interest and *act-1*. The level of expression in a transgenic strain compared to a control strain is the difference in the normalized number of cycles, with one cycle being equivalent to two-fold difference in expression.

### Paraquat resistance

Assays were performed in triplicate as previously described [48]. Briefly, four day old adult hermaphrodites were immersed in S-basal media containing 50 mM or 200 mM of paraquat. The number of dead worms was scored every hour by touch-provoked movement until all worms were dead. For each strain, median survival was determined by plotting Kaplan Meier survival curves containing 150 worms.

### ATP measurement

About 200 L4 hermaphrodites were collected, washed four times with S-basal buffer in an eppendorf tube, boiled for 20 minutes and quickly frozen in −80°C. All samples were processed on the same day. A Roche ATP Bioluminscent HSII kit was used to measure ATP concentrations, which measures bioluminescence emitted by the ATP-dependant oxidation of D-luciferin catalyzed by luciferase. ATP concentrations were determined using a standard curve derived from bioluminescence of known ATP concentrations (HSII kit). A Wallac 1420 multilabel counter luminometer (Victor2, Perkin Elmer) was used to measure levels of bioluminescence. A BioRad protein assay kit was used to measure protein concentrations using a Beckman Coulter DU 640 spectrophotometer. ATP concentrations were normalized to absolute protein concentrations. Each assay was repeated in triplicate, and the average ATP concentration and SD were calculated.

### Solid dietary restriction assay

The sDR method was performed as described in [49] with slight modifications. Overnight cultures of *E. coli* OP50 were grown at 37°C and collected by centrifugugation at 3,000 rpm for 30 minutes (Sorvall Legend RT) to collect bacterial cells. DR plates were prepared by adding 0.75×10^8^ cfu of OP50 and *ad libitum* (AL) plates were prepared by adding 0.75×10^11^ cfu of OP50 to NGM-FUDR plates. Worms were grown on NGM plates and synchronized hermaphrodites were transferred overnight to fresh NGM plates with OP50 and 30 µM of FUdR in order to prevent growth of progeny. Day 1 adult animals were then transferred to DR or AL plates. To maintain bacterial concentration, worms were transferred to fresh DR or AL plates every other day.

### Detection of carbonylated proteins

Oxidative damage was assessed using an Oxyblot assay kit (Millipore) to detect carbonylated proteins as previously described [50]. About 100 worms synchronized at day 1 or day 14 of adulthood were collected, washed twice with M9 buffer and boiled for 20 min in lysis buffer [50]. Carbonyl groups were derivatized to 2,4-dinitrophenylhydrazone (DNP-hydrazone), and were then detected by Western blotting with a DNP-specific antibody. Total protein levels in the extract were measured by nanodrop and 9 mg of protein lysate was loaded in each lane. Quantification of carbonylated proteins was achieved by taking the ratio of DNP staining to tubulin staining. Levels of carbonylated protein were compared in three independent samples of one day old and 14 day old adult worms.

### Quantification of *sod-3::mCherry* fluorescence

Fluorescence images of *sod-3::mCherry* were taken as described [30]. Briefly, 10 age-synchronized worms at day 9 of adulthood were transferred to 1 mM aldicarb-NGM plates for 2–3 hours to induce paralysis [51]. Worms were then photographed using a 20× lens on a Zeiss AxioPlan Fluorescent Microscope. Levels of mCherry expression (in the head and the first two pairs of intestinal cells) were analyzed using ImageJ [30]. For any given comparison, all pictures were taken on the same day with the same microscope settings. Results from three independent sets of 10 worms were used to calculate the average expression level and SD.

## Supporting Information

Figure S1 Phylogenetic trees of uncoupling protein and lysozyme genes. a. Uncoupling proteins. *C. elegans misc-1* is the closest to *C. elegans ucp-4* and is shown as a reference. Shown are phyogenetic trees for all uncoupling protein genes from the genomes of *C. elegans, D. melanogaster, D. rerio, M. musculus* and *H. sapiens*. *C. elegans* has one uncoupling protein gene (*ucp-4*) and the *ucp2* family is found only in vertebrates. b. Lysozyme tree. *C. elegans uaf-1* is used as a reference, being the gene closest to *lys-1*. *C. elegans* lysozyme genes belong to a family termed gh25 found mainly in unicellular microbes. *D. rerio lyz* (and other animal lysozyme genes in the tree) belong to the *lys* family which includes only animals but not *C. elegans*. Phylogenetic trees were generated using Muscle (Multiple Sequence Comparison by Log- Expectation) sequence alignment tool.
(TIF)

Figure S2 Brood size measurements of long-lived transgenic worms. Brood size was determined by counting the total number of progeny from a single hermaphrodite. Shown is the average and SEM for eight animals. y-axis shows the total number of progeny from individual hermaphrodites. Each bar represents transgenic worms expressing the corresponding gene. Control refers to worms expressing *unc-119(+); sod-3:mCherry*. The control bar represents an average of three independent lines. The brood size is not significantly smaller for worms expressing *C. elegans sod-1* or *lmp-2* and for worms expressing *D. rerio sod1*, *ucp2* or *lyz*. Wild type worms (N2) have a brood size of 295±25. *Ce* – *C. elegans*, *Dr* – *D. rerio*. * indicates strains with smaller brood size (p<0.05).
(TIF)

Table S1 Genes tested in this study.
(DOC)

Table S2 Lifespan of transgenic worms generated in this study.
(DOC)

Table S3 Additional data for genes that extend lifespan.
(DOC)

Table S4 Verification of transgene expression by RT–PCR.
(DOC)

Table S5 Summary of data from experiments on *B. subtilis*.
(DOC)

Table S6 Summary of sDR experiments.
(DOC)

Table S7 Lifespan extension is modular—summary of data for engineered strains.
(DOC)

Video S1 10-day-old control worm.
(WMV)

Video S2 18-day old-control worm.
(WMV)

Video S3 40-day-old quadruple longevity mutant.
(WMV)
