# Supplementary figures and images for: An Engineering Approach to Extending Lifespan in C. elegans
Source: PLoS Genet. 2012 Jun 21;8(6):e1002780. doi: 10.1371/journal.pgen.1002780 (PMC3380832; doi:10.1371/journal.pgen.1002780)

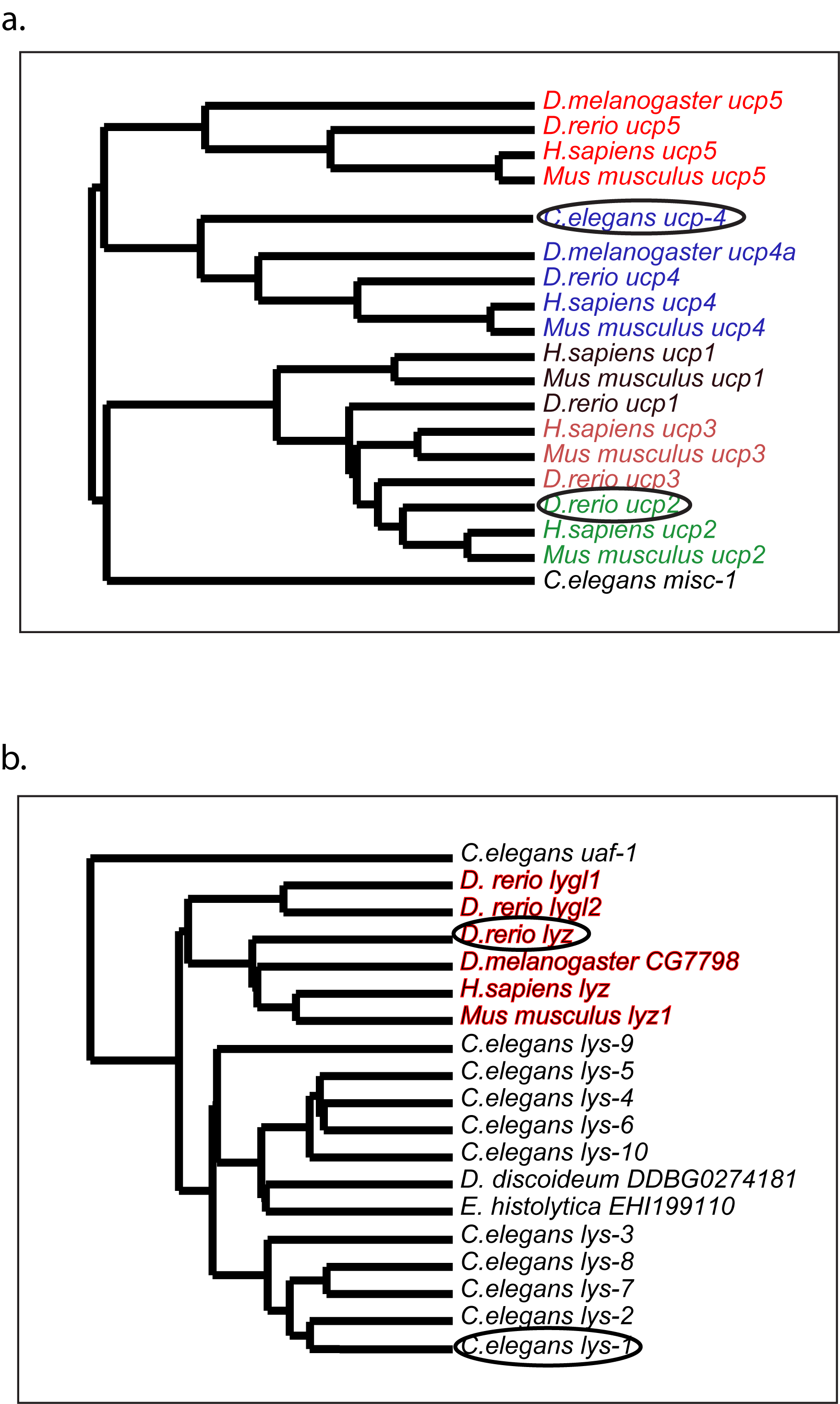

Supplement: Figure S1 — Phylogenetic trees of uncoupling protein and lysozyme genes. a. Uncoupling proteins. C. elegans misc-1 is the closest to C. elegans ucp-4 and is shown as a reference. Shown are phyogenetic trees for all uncoupling protein genes from the genomes of C. elegans, D. melanogaster, D. rerio, M. musculus and H. sapiens. C. elegans has one uncoupling protein gene (ucp-4) and the ucp2 family is found only in vertebrates. b. Lysozyme tree. C. elegans uaf-1 is used as a reference, being the gene closest to lys-1. C. elegans lysozyme genes belong to a family termed gh25 found mainly in unicellular microbes. D. rerio lyz (and other animal lysozyme genes in the tree) belong to the lys family which includes only animals but not C. elegans. Phylogenetic trees were generated using Muscle (Multiple Sequence Comparison by Log- Expectation) sequence alignment tool. (TIF) [file pgen.1002780.s001.tif]

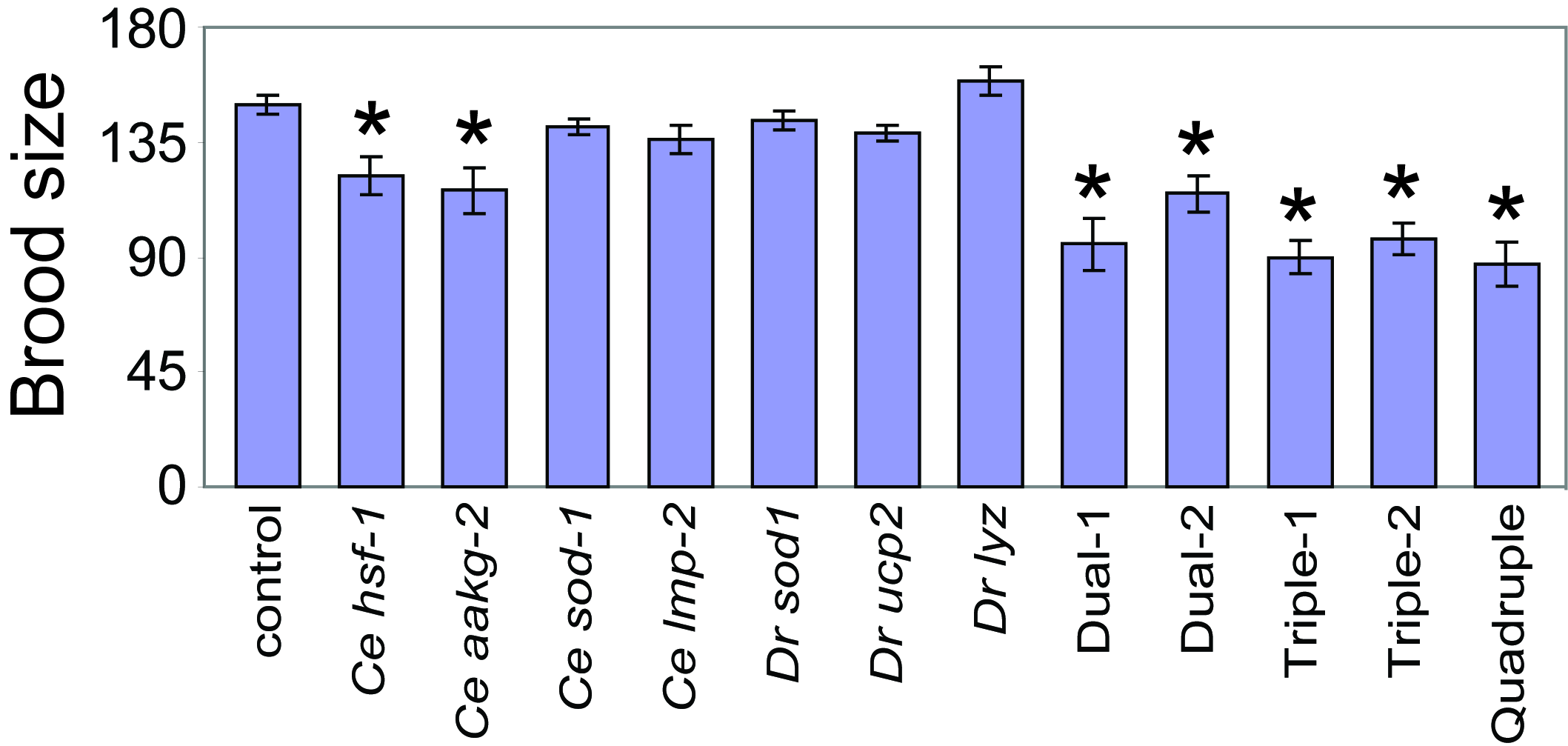

Supplement: Figure S2 — Brood size measurements of long-lived transgenic worms. Brood size was determined by counting the total number of progeny from a single hermaphrodite. Shown is the average and SEM for eight animals. y-axis shows the total number of progeny from individual hermaphrodites. Each bar represents transgenic worms expressing the corresponding gene. Control refers to worms expressing unc-119(+); sod-3:mCherry. The control bar represents an average of three independent lines. The brood size is not significantly smaller for worms expressing C. elegans sod-1 or lmp-2 and for worms expressing D. rerio sod1, ucp2 or lyz. Wild type worms (N2) have a brood size of 295±25. Ce – C. elegans, Dr – D. rerio. * indicates strains with smaller brood size (p<0.05). (TIF) [file pgen.1002780.s002.tif]
