# Supplementary material for: An Engineering Approach to Extending Lifespan in C. elegans
Source: PLoS Genet. 2012 Jun 21;8(6):e1002780. doi: 10.1371/journal.pgen.1002780 (PMC3380832; doi:10.1371/journal.pgen.1002780)
Supplement: Table S1 — Genes tested in this study. (DOC) [file pgen.1002780.s003.doc]

**Table S1**. Genes tested in this study.

| **Gene Name** | **Role** |
| --- | --- |
| *hsf-1^a,d^, daf-16^a^*  *aakg-2(sta2)^a,d^*  *sod-1^a,d^* | Regulates stress response  Senses ATP : AMP ratio  Reduces O_2_^-^ |
| *uba-1^a^*  *lmp-2^a,d^* | Increases protein turn-over  Induces chaperone-mediated autophagy |
| *sod1^b,d^*  *foxo3a^b^*  *msra^c^*  *psmb1^b^*  *aldh2^c^* | Reduces O_2_^-^  Regulates stress response  Repairs oxidized proteins  Increases protein turn-over  Oxidizes aldehydes |
| *ucp-4^a^, ucp2^b,d^*  *lys-1^a^, lyz^b,d^*  *lipc^b^* | Reduces proton gradient  Lyses bacterial cell walls  Reduces fat storage |

^a^*C. elegans*; ^b^*Danio rerio*; ^c^*Homo sapiens*; ^d^transgene extends lifespan

Table S1

Genes tested in this study and their corresponding roles.
