# Supplementary material for: An Engineering Approach to Extending Lifespan in C. elegans
Source: PLoS Genet. 2012 Jun 21;8(6):e1002780. doi: 10.1371/journal.pgen.1002780 (PMC3380832; doi:10.1371/journal.pgen.1002780)
Supplement: Table S2 — Lifespan of transgenic worms generated in this study. (DOC) [file pgen.1002780.s004.doc]

**Table S2**. Lifespan of transgenic worms generated in this study^a^.

| **upstream region** | **Gene name** | **Median lifespan change (%)^b^** | ***gaEx*** | **Strain** |
| --- | --- | --- | --- | --- |
| *sod-3* | *Dr sod-1* | -7; 10; -1  5; 7 | NA  NA | NA  NA |
| *sod-1* | *Dr sod-1* | 25; 30; 20  20; 27; 28 | *gaEx190*  *gaEx191* | SD1656  SD1657 |
| *sod-1* | *Ce sod-1* | 15; 30; 30 | *gaEx204* | SD1820 |
| *msra-1* | *Hs msra* | 15; 5  5; -4 | NA  NA | NA  NA |
| *sod-3* | *Hs msra* | 5; -5  0; -4 | NA  NA | NA  NA |
| *ucp-4* | *Dr ucp2* | 40; 47; 45  33; 40; 32 | NA  *gaEx192* | NA  SD1658 |
| *gpa-4* | *Dr ucp2* | 15; 10 | NA | NA |
| *sur-5* | *Dr ucp2* | -22; - 19 | NA | NA |
| *ucp-4* | *Ce ucp-4* | 0; -4  5; -8 | NA  NA | NA  NA |
| *aakg-2* | *aakg-2(sta2)* | 40; 50; 54  52; 38 | *gaEx207 gaEx208* | SD1823  SD1824 |
| *alh-1* | *Hs aldh2* | -5; | gaEx209 | SD1825 |
| *ges-1* | *Dr lipc* | -50; -30 | NA | NA |
| *hsf-1* | *Ce hsf-1* | 34; 45; 32  31; 35; 24 | *gaEx206* | SD1822 NA |
| *sod-3* | *Dr foxo3a* | 6; 10  -10; -4 | NA  NA | NA  NA |
| *sod-3* | *Ce daf-16* | -5; -12 | NA | NA |
| *lmp-2* | *Ce lmp-2* | 35; 36; 28  35 | *gaEx193 gaEx205* | SD1659  SD1821 |
| *uba-1* | *Ce uba-1* | 10; 15 | *gaEx210* | SD1826 |
| *pbs-6* | *Dr psmb1* | -50; | NA | NA |
| *ges-1* | *Ce lys-1* | -13; -18 | NA | NA |
| *lys-1* | *Ce lys-1* | 0; 10  -5; 0 | NA  NA | NA  NA |
| *lys-1* | *Dr lyz* | 23; 35; 26 | *gaEx189* | SD1655 |
| Dual-1 | [*Ce aakg-2(sta2); Dr ucp2*] | 78; 89; 68; 87; 75; 85  84 ; 73; 94; 94; 69; 88 | *gaEx214*  *gaEx215* | SD1901 |
| Dual-2 | [*Ce hsf-1; Dr lyz*] | 67; 53; 58;  58; 63; 50 | *gaEx216*  *gaEx217* | SD1902 |
| Triple-1 | [*Ce aakg-2(sta2); Dr ucp2; Dr lyz*] | 115; 93; 107    95; 105; 91 | *gaEx218*  *gaEx219* | SD1903 |
| Triple-2 | [*Ce hsf-1; Dr lyz; Ce aakg-2(sta2)*] | 85; 104; 87  95; 75; 82 | *gaEx220*  *gaEx221* | SD1904 |
| Quadruple | [*Ce hsf-1; Dr lyz; Ce aakg-2(sta2); Dr ucp2*] | 139; 148; 118  133; 105; 134 | *gaEx222*  *gaEx223* | SD1905 |

^a^at least 80 animals were counted in each experiment; ^b^change in median lifespan of animals compared to control lifespan (17-19 days) in independent experiments.

Table S2

Lifespan of transgenic worms generated in this study.
