# Supplementary material for: An Engineering Approach to Extending Lifespan in C. elegans
Source: PLoS Genet. 2012 Jun 21;8(6):e1002780. doi: 10.1371/journal.pgen.1002780 (PMC3380832; doi:10.1371/journal.pgen.1002780)
Supplement: Table S3 — Additional data for genes that extend lifespan. (DOC) [file pgen.1002780.s005.doc]

**Table S3**. Additional data for genes that extend lifespan.

| **genotype** | **strain** | **lifespan increase (%)^a,b^** | **number of animals** | **number of control animals** |
| --- | --- | --- | --- | --- |
| *Ce hsf-1* | SD1822  NA | 34^*^  45  32  31  35  24 | 84  93  79  100  82  85 | 80  85  82  93  88  87 |
| *Ce aakg-2(sta2)* | SD1823  SD1824 | 50^*^  40  54  52  38 | 81  83  78  79  90 | 81  79  82  87  84 |
| *Ce sod-1* | SD1820 | 30^*^  15  30 | 98  78  84 | 89  87  80 |
| *Ce lmp-2* | SD1659  SD1821 | 35^*^  36  28  35 | 80  85  82  95 | 91  82  86  92 |
| *Dr sod1* | SD1656  SD1657 | 25^*^  20  30  20  27  28 | 82  84  90  77  87  81 | 87  81  87  87  91  95 |
| *Dr ucp2* | SD1658  NA | 45^*^  47  40  33  40  32 | 80  95  85  78  85  88 | 80  85  84  85  86  86 |
| *Dr lyz* | SD1655 | 26^*^,  23  35 | 88  95  82 | 79  85  88 |

^a^median percentage increase in lifespan. All assays in this Table showed p < 0.01 determined by log-rank statistics. ^b^Control lifespan varies between 17 to 19 days; ^*^refers to the lifespan curves shown in Figure 1; 83 animals were scored in Figure 1f for the *Ce ucp-4* transgenic strain; 92 animals were scored in Figure 1g for the *Ce lys-1* transgenic strain.

Table S3

Additional data for transgenes that extend lifespan, including number of assays for each line and number of animals per assay.
