# Supplementary material for: An Engineering Approach to Extending Lifespan in C. elegans
Source: PLoS Genet. 2012 Jun 21;8(6):e1002780. doi: 10.1371/journal.pgen.1002780 (PMC3380832; doi:10.1371/journal.pgen.1002780)
Supplement: Table S4 — Verification of transgene expression by RT–PCR. (DOC) [file pgen.1002780.s006.doc]

**Table S4**. Verification of transgene expression by RT-PCR.

| **Gene** | **RNA expression**  **(fold change)^*^** |
| --- | --- |
|  |  |
| *Ce hsf-1* | 5.6 ± 0.9 |
| *Ce aakg-2(sta2)* | 9.2 ± 1.8 |
| *Ce sod-1* | 10.5 ± 2.4 |
| *Ce lmp-2* | 18.4 ± 2.5 |
| *Dr sod-1* | 1.0± 0.25^**^ |
| *Dr ucp2* | 1.0 ± 0.15^**^ |
| *Dr lyz* | 1.0 ± 0.17^**^ |
|  |  |
| Dual-1  [*Ce aakg-2(sta2); Dr ucp2*] | 7.1 ± 1.1, 1.5 ± 0.25^**^ |
| Dual-2  [*Ce hsf-1; Dr lyz*] | 4.1 ± 0.7, 1.1 ± 0.2^**^ |
|  |  |
| Triple-1  [*Ce aakg-2(sta2); Dr ucp2; Dr lyz*] | 5.8 ± 0.6, 0.5 ± 0.07^**^, 0.7 ± 0.1^**^ |
| Triple-2  [*Ce hsf-1; Dr lyz; Ce aakg-2(sta2)*] | 6.3 ± 0.8, 1.1 ± 0.05^**^, 8.3 ± 1.2 |
| Quadruple  [*Ce hsf-1; Dr lyz; Ce aakg-2(sta2); Dr ucp2*] | 4.7±0.8, 0.5±0.09^**^, 4.9±0.5, 0.5±0.1^**^ |

*fold change ± SD with respect to control strain (mean of three independent quantitative RT-PCR experiments). ^**^ Expression levels of *D. rerio* genes relative to expression levels measured in single-component strains.

Table S4

Expression levels of the various transgenes in transgenic worms used in this study, as measured by RT-PCR.
