# Supplementary material for: An Engineering Approach to Extending Lifespan in C. elegans
Source: PLoS Genet. 2012 Jun 21;8(6):e1002780. doi: 10.1371/journal.pgen.1002780 (PMC3380832; doi:10.1371/journal.pgen.1002780)
Supplement: Table S5 — Summary of data from experiments on B. subtilis. (DOC) [file pgen.1002780.s007.doc]

**Table S5**. Summary of data from experiments on *B. subtilis*.

| **food source** | **genotype** | **Lifespan increase (%)^a,b^** | **number of animals** | **number of control animals** |
| --- | --- | --- | --- | --- |
| *E. coli* | *Dr lyz* | 30^*^  25  27  25  22  32 | 84^*^  92  87  80  77  86 | 83^*^  79  82  95  80  88 |
| *B. subtilis* | *Dr lyz* | none | 99^*^  78  82  88  87  90 | 89^*^  77  85  85  84  88 |

^a^median percentage increase in lifespan (p < 0.01 determined by log-rank statistics); ^b^control lifespan varies between 17 to 19 days; ^*^refers to lifespan curves that are also shown in Figure 2.

Table S5

Summary of data from experiments on *B. subtilis*.
