# Supplementary material for: An Engineering Approach to Extending Lifespan in C. elegans
Source: PLoS Genet. 2012 Jun 21;8(6):e1002780. doi: 10.1371/journal.pgen.1002780 (PMC3380832; doi:10.1371/journal.pgen.1002780)
Supplement: Table S6 — Summary of sDR experiments. (DOC) [file pgen.1002780.s008.doc]

**Table S6**. Summary of sDR experiments

| **genotype** | **Lifespan increase under sDR (%)** | **Number of**  **Animals under AL** | **Number of control**  **Animals under sDR** | **p-value** |
| --- | --- | --- | --- | --- |
| control | 18 ^*^  22 | 88  95 | 99  83 | 6*10^-3^  5*10^-3^ |
| *Dr ucp2* | ns^*^  ns | 85  81 | 78  80 | >0.05  >0.05 |
| *Ce aakg-2(sta2)* | ns^*^  ns | 89  79 | 83  85 | >0.05  >0.05 |
| Dual-1 | ns ^*^  ns | 87  85 | 82  92 | >0.05  >0.05 |

^*^refers to the lifespan curves shown on figure 3. ns – not significant.

Table S6

Summary of sDR experiments, including lifespan extension and number of animals in each assay.
