# Supplementary material for: An Engineering Approach to Extending Lifespan in C. elegans
Source: PLoS Genet. 2012 Jun 21;8(6):e1002780. doi: 10.1371/journal.pgen.1002780 (PMC3380832; doi:10.1371/journal.pgen.1002780)
Supplement: Table S7 — Lifespan extension is modular—summary of data for engineered strains. (DOC) [file pgen.1002780.s009.doc]

**Table S7**. Lifespan extension is modular - summary of data for engineered strains.

| **Genotypes^a^** | **Lifespan increase (%)^b^** | **Number of animals** | **number of control animals** |
| --- | --- | --- | --- |
| *Dr lyz*  *Dr ucp2*  *Ce aakg-2(sta2)*  Dual-1  Triple-1 | 26 ^*^; 23; 25  35 ^*^; 38; 39  44 ^*^; 47; 45  78 ^*^; 89; 68  107 ^*^; 115; 93 | 85; 84; 84  91; 90; 82  81; 87; 91  81; 82; 88  87; 97; 86 | 88; 91; 83 |
| *Dr lyz*  *Ce hsf-1*  *Ce aakg-2 (sta2)* Dual-2  Triple-2 | 22 ^*^; 26; 20  32 ^*^; 35; 31  44 ^*^; 44; 42  58 ^*^; 67; 53  87; 85; 104 | 95; 84; 84  93; 90; 82  84; 87; 91  88; 82; 88  87; 97; 86 | 91; 91; 83 |
| Dual-1  Triple-1  Quadruple | 82 ^*^; 73; 88  117 ^*^; 105; 97  139 ^*^; 148; 118 | 99; 92; 89  93; 80; 91  112; 87; 101 | 101; 81; 79 |

^a^Each block refers to parallel lifespan experiments demonstrating a progressive increase in lifespan with an increase in the number of components; each column represents parallel lifespan experiments comparing the corresponding genotypes. Each row represents three independent repeats. ^b^Shown is the median percentage increase in lifespan. The control median lifespan is 17-19 days.  ^*^refers to the lifespan curves shown in Figure 5.

Table S7

Lifespan extension is modular - summary of data for engineered strains.
